# Tyrosine phosphatase STEP_61_ in human dementia and in animal models with amyloid and tau pathology

**DOI:** 10.1186/s13041-023-00994-3

**Published:** 2023-01-13

**Authors:** Deonne Taylor, Andrew Kneynsberg, Marloes van Roijen, Jürgen Götz

**Affiliations:** 1 Clem Jones Centre for Ageing Dementia Research, Queensland Brain Institute, The University of Queensland, St. Lucia Campus, Brisbane, QLD Australia; 2 New South Wales Brain Bank, The University of Sydney, NSW Sydney, Australia

**Keywords:** Alzheimer’s disease, Amyloid-β, Frontotemporal dementia, Striatal-enriched tyrosine phosphatase 61 (STEP_61_), Microtubule-associated protein tau, Transgenic mice

## Abstract

Synaptic degeneration is a precursor of synaptic and neuronal loss in neurodegenerative diseases such as Alzheimer’s disease (AD) and frontotemporal dementia with tau pathology (FTD-tau), a group of primary tauopathies. A critical role in this degenerative process is assumed by enzymes such as the kinase Fyn and its counterpart, the phosphatase striatal-enriched tyrosine phosphatase 61 (STEP_61_). Whereas the role of Fyn has been widely explored, less is known about STEP_61_ that localises to the postsynaptic density (PSD) of glutamatergic neurons. In dementias, synaptic loss is associated with an increased burden of pathological aggregates. Tau pathology is a hallmark of both AD (together with amyloid-β deposition) and FTD-tau. Here, we examined STEP_61_ and its activity in human and animal brain tissue and observed a correlation between STEP_61_ and disease progression. In early-stage human AD, an initial increase in the level and activity of STEP_61_ was observed, which decreased with the loss of the synaptic marker PSD-95; in FTD-tau, there was a reduction in STEP_61_ and PSD-95 which correlated with clinical diagnosis. In APP23 mice with an amyloid-β pathology, the level and activity of STEP_61_ were increased in the synaptic fraction compared to wild-type littermates. Similarly, in the K3 mouse model of FTD-tau, which we assessed at two ages compared to wild-type, expression and activity of STEP_61_ were increased with ageing. Together, these findings suggest that STEP contributes differently to the pathogenic process in AD and FTD-tau, and that its activation may be an early response to a degenerative process.

**Supplementary Information:**

The online version contains supplementary material available at 10.1186/s13041-023-00994-3.

Alzheimer’s disease (AD) is the most prevalent form of all dementias. This neurodegenerative disorder is characterized by synaptic and neuronal loss in defined brain areas. The disease is further characterized by aggregates of extracellular amyloid-β-containing plaques and intracellular tau-containing tangles. AD shares tau pathology with frontotemporal dementia with tau (FTD-tau) which exists as various subtypes, including Pick’s disease (PiD), corticobasal degeneration (CBD) and progressive supranuclear palsy (PSP) [1]. Whereas neuropsychological assessment is used for clinical diagnosis of AD and FTD-tau, the definitive diagnosis rests on the assessment of the distribution and severity of tau and amyloid-β deposition in the post mortem brain [2, 3]. As the aggregation process is believed to initiate or at least, in part, drive the degenerative process, transgenic mouse models expressing tau or amyloid-β have contributed to our understanding of the degenerative process leading to synaptic dysfunction in dementias [4].

The synapse and its connectivity are known to be modulated by the selective activity of phosphotransferases, with Fyn kinase upregulating synaptic strength, and the striatal-enriched tyrosine phosphatase 61 (STEP_61_) downregulating signalling and synaptic connectivity [5, 6]. Substrates of STEP_61_ include the kinases ERK, Pyk2 and Fyn, as well as the glutamate receptors NMDAR and AMPAR [7]. This combined role allows STEP_61_ to dampen the response to extracellular signals through the ERK cascade as well as directly initiating internalisation of glutamate receptors [7]. STEP_61_ dysfunction is associated with many neurological disorders including developmental disorders (autism spectrum disorder and Fragile X syndrome), psychiatric conditions (schizophrenia, anxiety-related and depressive disorders), and neurodegenerative diseases (AD and Parkinson’s disease) [8]. Understanding STEP_61_ may thus be critical for paving the way for new therapies.

In this study, we first examined STEP_61_ in cases of AD and mild cognitive impairment (MCI), to determine how STEP_61_ expression and activity are altered over the progression of AD (Additional file 1: Materials and Methods; Table S1). Previous observations of STEP_61_ in AD reported increased STEP_61_ activity compared to healthy controls [9]. We, however, did not detect a similar increase between clinically diagnosed human cohorts, as neither the expression level of STEP_61_ nor its activity changed significantly in MCI or AD compared to cognitively normal controls (Additional file 1: Fig. S1A). Not surprisingly, the clinically diagnosed controls displayed a range of Braak stages, ranging from 0–III/IV. To further investigate this discrepancy and the variability in the clinically diagnosed control group, we established that STEP_61_ phosphorylation and expression levels were not correlated with post mortem delay (Additional file 1: Fig. S2), and then we sorted the cohort by pathological criteria based on the Braak staging of tau pathology. These comparisons revealed a significantly increased STEP_61_ activity at Braak stages I–II, compared to stage 0 or stages V/VI, as well as an increased STEP_61_ expression at stages I–II compared to V/VI (Fig. 1A). This finding indicates that a vacillating behaviour of STEP_61_ occurs over the course of AD progression and the accumulation of phosphorylated tau and high molecular weight species of tau. Interestingly, we observed a similar pattern of increase followed by a decline in the postsynaptic density protein (PSD-95) expression in the human samples sorted by Braak staging (Fig. 1A; Additional file 1: Fig. S3).

**Fig. 1:**
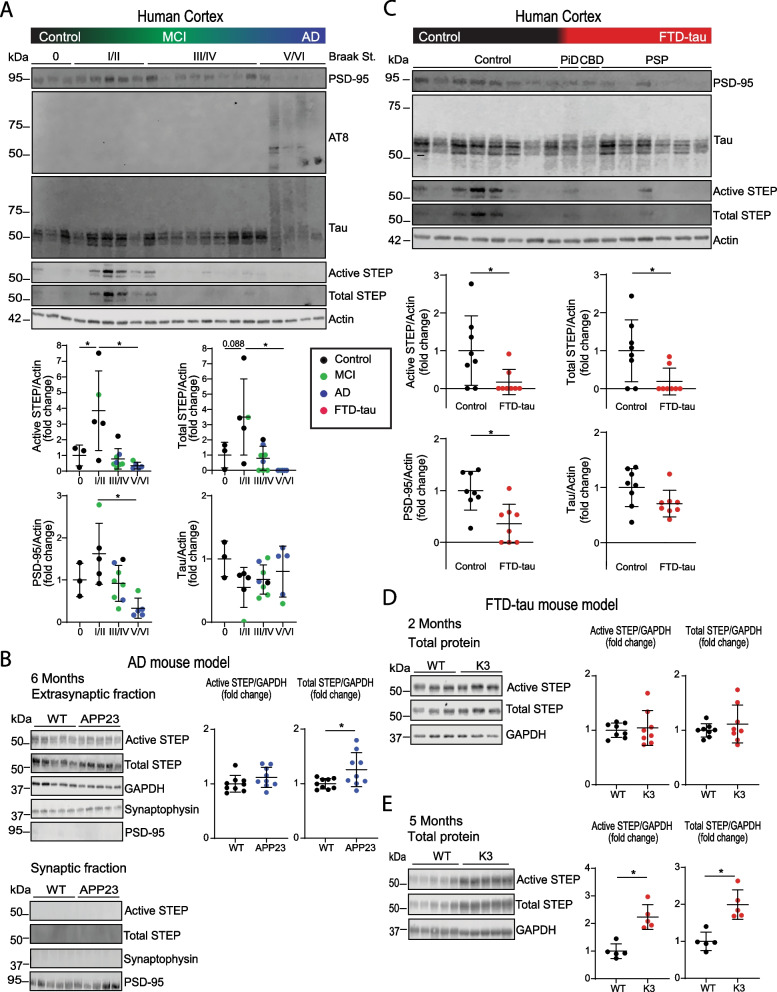
Modulation of STEP_61_ in human dementias and representative mouse models. **A** Immunoblot of RIPA-extracted tissue from the superior frontal cortex of patients diagnosed with AD, MCI and healthy controls and ordered by Braak stages. Of note, the controls in panels **A** and **C** are the same samples, only that in A they are grouped according to their Braak stage (with most controls being clinically normal displaying advanced Braak stages upon autopsy), whereas in **C** all controls are combined given that for FTD-tau (PiD, CBD and PSP) the Braak staging method is not available. Protein levels were normalised to actin. One way ANOVA multiple comparisons correction was performed on each data set; *p < 0.05; active STEP p = 0.0031, total STEP p = 0.0036, PSD-95, p = 0.0027, tau, p = 0.5730. **B** Immunoblot and quantification of extrasynaptically enriched fractions from 6-month-old APP23 mice (n = 9) and wild-type (WT) littermate controls (n = 9). Quantification of active and total STEP_61_ normalised to GAPDH; unpaired t-test, active STEP p > 0.5, and total STEP p = 0.0315. **C** Immunoblot of RIPA-extracted tissue from the superior frontal cortex of FTD-tau (n = 8) ordered by disease and PSP severity (Braak staging) and controls (n = 8). AT8-positive tau was not detectable in FTD-tau patients. Protein levels were normalised for β-actin. One way ANOVA with multiple comparisons correction performed on each data set; active STEP p = 0.0299, total STEP p = 0.0213, PSD-95, p = 0.0043, tau p = 0.0708. **D** Immunoblot of the RIPA fraction of cortical tissue from 2-month-old K3 mice (n = 8) and WT littermate controls (n = 8). Quantification of total and active STEP_61_ normalised to GAPDH; unpaired t-test, active STEP p = 0.1764, total STEP p = 0.0523. **E** Immunoblot of the RIPA fraction of cortical tissue from 5-month-old K3 mice (n = 5) and WT littermates (n = 5) probed for total and active STEP_61_. Quantification of active and total STEP_61_ normalised to GAPDH; unpaired t-test, active STEP p = 0.0007, total STEP p = 0.0015 (see Additional file 2: Statistical information)

We next examined the activation and expression of STEP_61_ in amyloid-depositing APP23 mice, finding no significant change in the total protein level in cortical lysates at 6 months of age. However, whereas STEP_61_ was virtually excluded from the synaptic fraction, isolation of the extrasynaptic fraction revealed a significant increase of STEP_61_ levels and activity compared to non-transgenic littermates (Fig. 1B; Additional file 1: Fig. S4). In addition to amyloid-β, we have previously observed a significant increase in tau protein at 6 months of age in the APP23 mouse model [10]. This model does not display synaptic or neuronal loss with the accumulation of amyloid pathology at 24 months of age, as reported by Boncristiano and colleagues [11].

We next analysed STEP_61_ in tissue from patients clinically diagnosed with different subtypes of FTD-tau (PiD, CBD and PSP, see Additional file 1: Table S1). Here, we used the same controls as in Fig. 1A, but grouped them together given that there are no Braak stages for FTD-tau. Compared to healthy controls, the expression and activity of STEP_61_ were significantly decreased in FTD-tau tissue (Fig. 1C). This decrease of STEP_61_ was observed together with a significant decrease in total PSD-95, reflecting synaptic loss in these patients (Fig. 1C; Additional file 1: Fig. S3). In contrast to MCI and AD patients, the analysis of FTD-tau patients by Braak staging did not reveal differences in STEP_61_ or PSD-95 levels (Additional file 1: Fig. S1B).

Patients suspected of FTD-tau diagnosis present with a range of clinical symptoms, and have complex pathological characterisations with multiple tau- and co-pathologies upon post mortem analysis [12]. Compared to the human pathology, the K3 mouse model exhibits a simpler presentation of tau and its accumulation. We have previously characterised the expression of tau in the cortex of 2- and 5-month-old K3 mice, revealing a significant increase in the expression of tau at 5 months of age [13]. When we examined cortical tissue from these mice, we found no significant changes in the level of STEP_61_ at 2 months of age (Fig. 1D). However, at 5 months of age, we observed an increase in the expression level and activity of STEP_61_, suggesting that the higher expression, accumulation, or prolonged presence of tau could increase STEP_61_ (Fig. 1E). A limitation of our study is that the K3 model cannot recapitulate the later stages of human disease, as the transgenic line has drifted since its generation and the mice experience no neuronal loss with age anymore despite their tau pathology [14]. This discrepancy from human pathophysiology limits the conclusions that can be drawn about human disease from what we see here in the K3 mouse.

Together, our findings indicate that STEP_61_ responds to pathological insults in a time- and localization-dependent manner. Observations in human patients and animal models suggest that an increase of STEP_61_ occurs early in the course of disease, before the overt loss of synapses. A decrease in STEP_61_ expression correlates with significant synaptic (and neuronal) loss as AD progresses. We also found changes in STEP_61_ and its activity in the synaptic compartment when amyloid-β initially accumulates, suggesting localised synaptic activation upon insult. Contrasting human FTD-tau to the mouse model of inherited tauopathy, the response of STEP_61_ to pathological tau likely depends on the severity of pathology and the type of tau that is driving dysfunction. Together, our data add to the multifaceted mechanisms of degeneration in human dementia and corresponding transgenic models. Further studies into STEP_61_ are warranted, to better understand how this enzyme is differentially regulated in primary and secondary tauopathies.

## Supplementary Information

**Additional file 1:** Materials and methods. **Methods.** Reagents and antibodies, human samples, experimental mice, tissue processing, sequential protein fractionation, subcellular fractionation, western blots, statistical analysis; **Materials.** Human tissue samples. **Table S1.** Human patient information for clinically diagnosed FTD, AD, MCI and healthy control patients. **Figure S1.** Changes in STEP61 in the cortex of AD and FTD patients. A Quantification of levels of STEP61, its activity, PSD-95 and tau normalised to actin in AD (n=6), MCI (n=7) and healthy control (n=8). Fold change of the healthy control. One-way ANOVA post-hoc multiple comparisons correction performed with Tukey’s test, active STEP p=0.1093, total STEP p=0.0919, PSD-95 p=0.1144, tau p=0.8275. B Quantification of levels of STEP61, its activity, PSD-95 and tau normalised to actin in FTD (n=8) and healthy control (n=8) cortex, plotted by Braak staging, fold change of healthy controls. Analysed with unpaired Student’s t-test used for each quantification, active STEP p=0.9947, total STEP p=0.9098, PSD-95 p=0.9312, tau p=0.7568. **Figure S2.** No correlation detected between post mortem delay and STEP61 activity or expression. To determine whether post mortem delay effected the phosphorylation state of STEP61, as dephosphorylation results in STEP61 activation. Long delays can result in degradation of proteins or dephosphorylation by endogenous phosphatases, however we observed no correlation between post mortem delay and STEP61 activation or expression or PSD-95 expression. Colour coded to show clinical diagnosis of patients. A Correlation between active STEP61 expression and delay (hrs) of all patients. Correlation analysis by Pearson’s correlation coefficient, all grouped R^2^= 0.01425. B Correlation between total STEP61 expression and delay (hrs) of all patients. Correlation analysis by Pearson’s correlation coefficient, all grouped R^2^= 0.04894. C Correlation between total PSD-95 expression and delay (hrs) of all patients. Correlation analysis by Pearson’s correlation coefficient, all grouped R^2^= 0.09331. **Figure S3.** Correlation detected between PSD-95 expression and STEP61 expression in AD and FTD cohorts. Correlation between the expression of total STEP and PSD-95 expression in AD and FTD human cohorts. Shows decrease in STEP61 expression correlates with reduced PSD-95 expression. Colour coded to show clinical diagnosis of patients. A Correlation between total STEP61 expression and PSD-95 of all AD (blue), MCI (green) and control (black) patients. Correlation analysis by Pearson’s correlation coefficient, R^2^= 0.5297. B Correlation between total STEP61 expression and PSD-95 of all FTD-tau (red) and control (black) patients. Correlation analysis by Pearson’s correlation coefficient, R^2^= 0.3604. **Figure S4.** Fractionation protocol quality was identified by extrasynaptic enriched synaptophysin and synaptic enriched PSD-95. Immunoblot of the extrasynaptic and synaptic fractions of 6-month-old APP23 mice (n=4) and their WT littermate controls (n=4). Extrasynaptic fractions were enriched for synaptophysin, whereas the synaptic fraction was enriched with the post-synaptic marker PSD-95.

**Additional file 2:** Statistical information.

## Data Availability

Additional materials, data, and detailed methods are included in the Additional file. All data supporting the findings of this study are available from the corresponding author on reasonable request.

## Abbreviations

AD: Alzheimer’s disease
CBD: Corticobasal degeneration
FTD-tau: Frontotemporal dementia with tau pathology
MCI: Mild cognitive impairment
PiD: Pick’s disease
STEP61: Striatal-enriched tyrosine phosphatase
PSD-95: Postsynaptic density protein 95
PSP: Progressive supranuclear palsy
SD: Standard deviation
