# Supplementary material for: Tyrosine phosphatase STEP61 in human dementia and in animal models with amyloid and tau pathology
Source: Mol Brain. 2023 Jan 13;16:6. doi: 10.1186/s13041-023-00994-3 (PMC9840288; doi:10.1186/s13041-023-00994-3)
Supplement: Supplementary file 1 — Additional file 1: Materials and methods. Methods. Reagents and antibodies, human samples, experimental mice, tissue processing, sequential protein fractionation, subcellular fractionation, western blots, statistical analysis; Materials. Human tissue samples. Table S1. Human patient information for clinically diagnosed FTD, AD, MCI and healthy control patients. Figure S1. Changes in STEP61 in the cortex of AD and FTD patients. A Quantification of levels of STEP61, its activity, PSD-95 and tau normalised to actin in AD (n=6), MCI (n=7) and healthy control (n=8). Fold change of the healthy control. One-way ANOVA post-hoc multiple comparisons correction performed with Tukey’s test, active STEP p=0.1093, total STEP p=0.0919, PSD-95 p=0.1144, tau p=0.8275. B Quantification of levels of STEP61, its activity, PSD-95 and tau normalised to actin in FTD (n=8) and healthy control (n=8) cortex, plotted by Braak staging, fold change of healthy controls. Analysed with unpaired Student’s t-test used for each quantification, active STEP p=0.9947, total STEP p=0.9098, PSD-95 p=0.9312, tau p=0.7568. Figure S2. No correlation detected between post mortem delay and STEP61 activity or expression. To determine whether post mortem delay effected the phosphorylation state of STEP61, as dephosphorylation results in STEP61 activation. Long delays can result in degradation of proteins or dephosphorylation by endogenous phosphatases, however we observed no correlation between post mortem delay and STEP61 activation or expression or PSD-95 expression. Colour coded to show clinical diagnosis of patients. A Correlation between active STEP61 expression and delay (hrs) of all patients. Correlation analysis by Pearson’s correlation coefficient, all grouped R2= 0.01425. B Correlation between total STEP61 expression and delay (hrs) of all patients. Correlation analysis by Pearson’s correlation coefficient, all grouped R2= 0.04894. C Correlation between total PSD-95 expression and delay (hrs) of all pa [file 13041_2023_994_MOESM1_ESM.docx]

**Tyrosine phosphatase STEP_61_**

**in human dementia and in animal models with amyloid and tau pathology**

Deonne Taylor^1^, Andrew Kneynsberg^1^, Marloes van Roijen^2^ & Jürgen Götz^1^

1. Clem Jones Centre of Ageing and Dementia Research, Queensland Brain Institute, The University of Queensland, St. Lucia Campus, QLD, Australia
2. New South Wales Brain Bank, The University of Sydney, Sydney, NSW, Australia

**MATERIALS AND METHODS**

**METHODS**

***Reagents and antibodies***

Anti-GAPDH antibody (#MAB374), anti-Actin (#MAB1501R), anti-PSD95 (#MABN68), anti-STEP (cell signalling, #4396), anti-non phospho STEP (#5659), anti-Tau (DAKO Tau) and anti-AT8 (#MN1020).

***Human samples***

Human brain samples were obtained from the Sydney Brain Bank and approved by the Human Ethics Committee for use in this study. Mildly cognitive impaired (MCI), Alzheimer’s disease (AD), frontotemporal dementia with tau (FTD-tau) and control tissue samples were from the superior frontal cortex. Control cases were defined clinically, i.e. with no cognitive decline detectable. MCI cases did not meet NIA Reagan AD criteria for AD, which requires diagnosis of both neurofibrillary tangles (Braak) and neuritic plaque (CERAD). FTD-tau cases were determined and differentiated based on published neuropathological consensus criteria, with histopathological assessment of both tau-positive astrocytes (astrocytic plaques/tufted astrocytes/ramified astrocytes) and/or tau-positive neurons (Pick bodies/globose tangles).

***Experimental Mice***

The following transgenic mice were used in the study, together with age-matched non-transgenic littermate controls: APP23 mice express the human APP gene together with the Swedish double mutation KM670/671NL under control of the murine Thy1 promoter. The mice are characterized by amyloid plaques throughout the cortex and hippocampus, synaptic and subtle neuronal loss, as well as learning and memory impairments. K3 mice are a model of primary tauopathy that express human 1N4R tau with the K369I mutation identified in patients with PiD, expressed under control of the murine Thy1.2 promoter. The mice are characterized by intraneuronal tau aggregates that predominantly resemble Pick-body like inclusions. Behaviourally, the mice are characterized by memory and motor deficits. All mice were maintained and bred under standard conditions, and all animal experiments were approved by the Animal Ethics Committee of the University of Queensland (Ethics #QBI/554/17).

***Tissue processing***

Mice were anesthetised by intraperitoneal injection of pentobarbital, followed by transcardial perfusion with 25 ml PBS. Brains were dissected and hemispheres separated. The left hemisphere was post-fixed in 4% PFA, and the right hemisphere was dissected into cortex, hippocampus and the remainder of the brain tissue, snap frozen and stored at -80°C.

***Sequential protein fractionation***

Sequential protein extraction of the right-brain dissected tissue was performed as follows: Cortices and hippocampi were separately homogenized in RAB buffer containing complete a phosphatase inhibitor cocktail (Invitrogen), and a protease inhibitor tablet (Roche). The tissue was homogenized using a tissue lyser for 6 minutes and using an increasing gauge needle for the hippocampus followed by a short sonication step (5s/5s). The lysate was then removed and labelled as total protein. Sonication was then followed by a centrifugation at 21,000g for 90 minutes to extract the RAB-soluble fraction. The pellet was then suspended in the same volume of RIPA buffer containing protease and phosphatase inhibitors, and was centrifuged at 21,000g for 90 minutes to obtain the RIPA-soluble fraction. Concentration of the fractions was determined by the BCA assay (Pierce). All extractions steps were performed at 4°C and the final aliquots and pellet were stored at -80°C until required.

***Subcellular fractionation***

Subcellular enrichment of the right-brain dissected tissue was performed as follows: Cortices and hippocampi were homogenized in sucrose buffer containing a phosphatase inhibitor cocktail (Invitrogen) and a protease inhibitor tablet (Roche) via a manual drill and by using increasing gauge needles. All buffers, including the wash buffer, contained protease and phosphatase inhibitors. Total protein was removed followed by a centrifugation at 1,000g for 10 minutes, then the S1 fraction was removed, leaving the nuclear pellet. The S1 fraction was then centrifuged at 14,000g for 20 minutes, yielding the S2 fraction (microsomes and cytosol) and the P2 fraction (crude synaptosomal membranes). The S2 fraction was removed as the cytosolic fraction, and the P2 fraction was re-suspended in wash buffer and centrifuged at 12,000g for 20 minutes. The resulting pellet was re-suspended in buffer A, containing 20 mM HEPES, 100 mM NaCl and 0.5% Triton-X-100 (pH 7.2) and then slowly rotated for 1 hour. Then, the sample was centrifuged at 12,000g for 20 minutes to yield the S3 (extrasynaptic) and P3 fractions. The P3 fraction was re-suspended in wash buffer and centrifuged as described above. This was then centrifuged at 12,000g for 20 minutes and re-suspended in buffer B, containing 20 mM HEPES, 0.15 mM NaCl, 1% Triton-X-100, 1% SDS, 1 mM DTT, and 1% deoxycholate pH 5, and then rotated slowly for 1 hour and centrifuged at 10,000g for 20 minutes to yield the S4 fraction (synaptic). All centrifugation steps were conducted at 4°C with all fraction aliquots and pellets being stored at -80°C until use.

***Western blots***

Total protein, detergent insoluble fractions and subcellular enriched protein samples (15-20 µg) were separated in a 10% SDS-page gel and transferred onto a nitrocellulose membrane using the Criterion blotter (Bio-Rad), and 20% methanol/20% glycine transfer buffer (pH 8.6). Membranes were blocked in TBS containing Odyssey Blocking Buffer (LI-COR) and incubated in primary antibodies overnight at 4°C followed by incubation with the secondary antibody for one hour at RT. Membranes were imaged on the LI-COR Odyssey scanner, detected bands were quantified using the Image Studio software (LI-COR) and normalized to GAPDH or β-actin for quantification. Human samples were pure cortical samples which only expressed the isoform STEP61, compared to the mouse samples which included whole cerebral cortex and expressed a high level of the isoform STEP46. This impeded the quantification of the β-actin, leading to use of GAPDH for protein quantification.

***Statistical analysis***

All data values are displayed as mean +/- SD. Data sets with two groups had Student’s t-tests applied. One-way ANOVA was used for comparisons of data with three groups or more. Post-hoc group multiple comparisons were performed with Tukey’s test. A value of p<0.05 for all data was considered significant.

**MATERIALS**

| Case Characterisation | Braak stage | Number of patients | Gender | Age  (years) | Disease duration (years) | *Post mortem* delay (hrs) |
| --- | --- | --- | --- | --- | --- | --- |
| Control | 0-Ⅲ | 8 | F | 86.8 ± 3.85 | 0 ± NA | 23.9 ± 6.58 |
| PiD | 0 | 1 | F | 82.0 | 12 | 25.0 |
| CBD | Ⅲ | 1 | F | 87.0 | 6 | 36.0 |
| PSP | Ⅰ-Ⅳ | 6 | F | 74.5 4.97 | 5.5 ± 2.95 | 17.3 ± 6.78 |
| MCI | Ⅱ-Ⅴ | 7 | F | 91.0 ± 6.73 | 1.1 ± 2.61 | 13.7 ± 11.5 |
| AD | Ⅳ-Ⅵ | 6 | F | 90.3 ± 14.07 | 7.8 ± 5.23 | 10.5 ± 6.28 |

**Supplementary Table 1. Human patient information for clinically diagnosed FTD, AD, MCI and healthy control patients.**


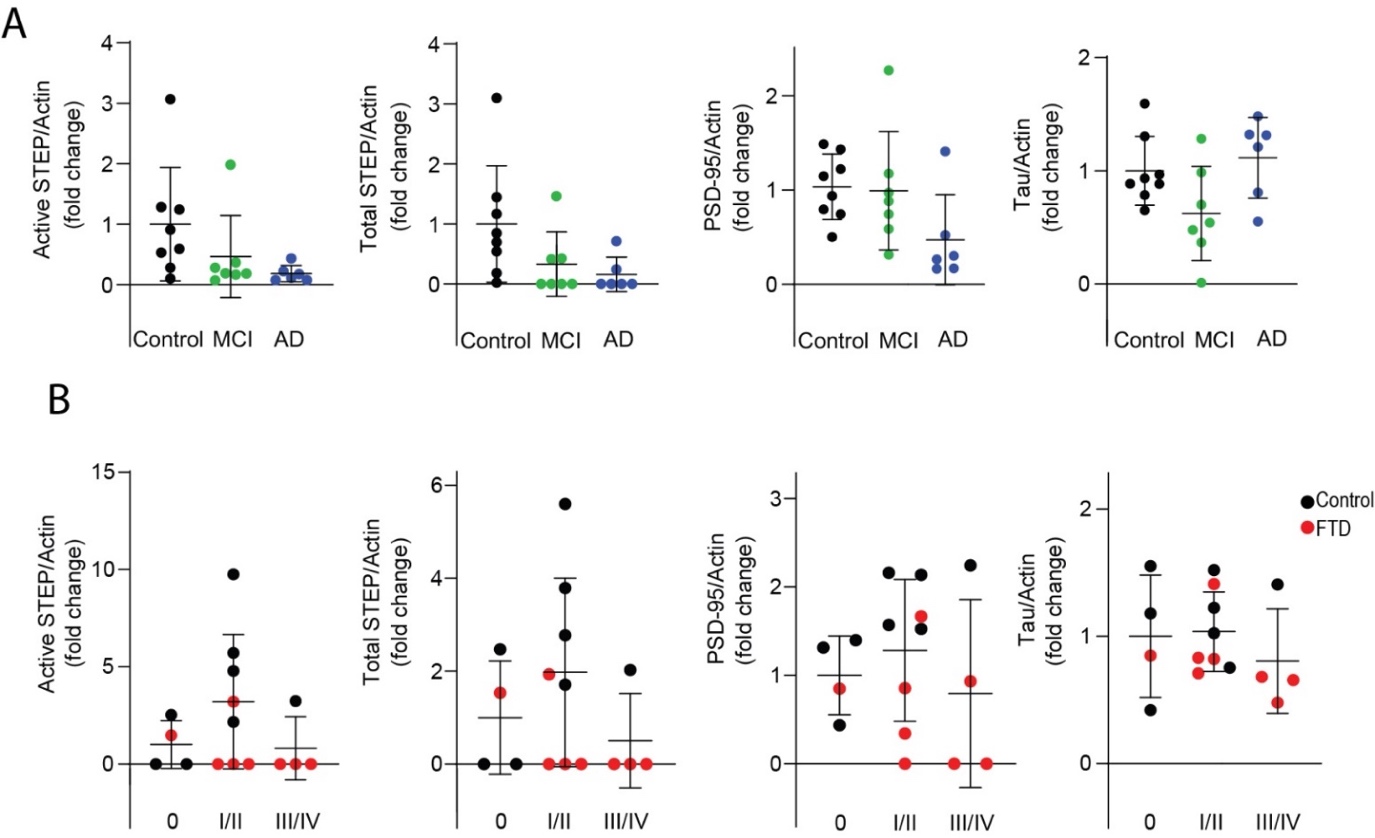


**Supplementary Figure 1. Changes in STEP_61_ in the cortex of AD and FTD patients.**

**A** Quantification of levels of STEP_61_, its activity, PSD-95 and tau normalised to actin in AD (n=6), MCI (n=7) and healthy control (n=8). Fold change of the healthy control. One-way ANOVA post-hoc multiple comparisons correction performed with Tukey’s test, active STEP p=0.1093, total STEP p=0.0919, PSD-95 p=0.1144, tau p=0.8275. **B** Quantification of levels of STEP_61_, its activity, PSD-95 and tau normalised to actin in FTD (n=8) and healthy control (n=8) cortex, plotted by Braak staging, fold change of healthy controls. Analysed with unpaired Student’s t-test used for each quantification, active STEP p=0.9947, total STEP p=0.9098, PSD-95 p=0.9312, tau p=0.7568.


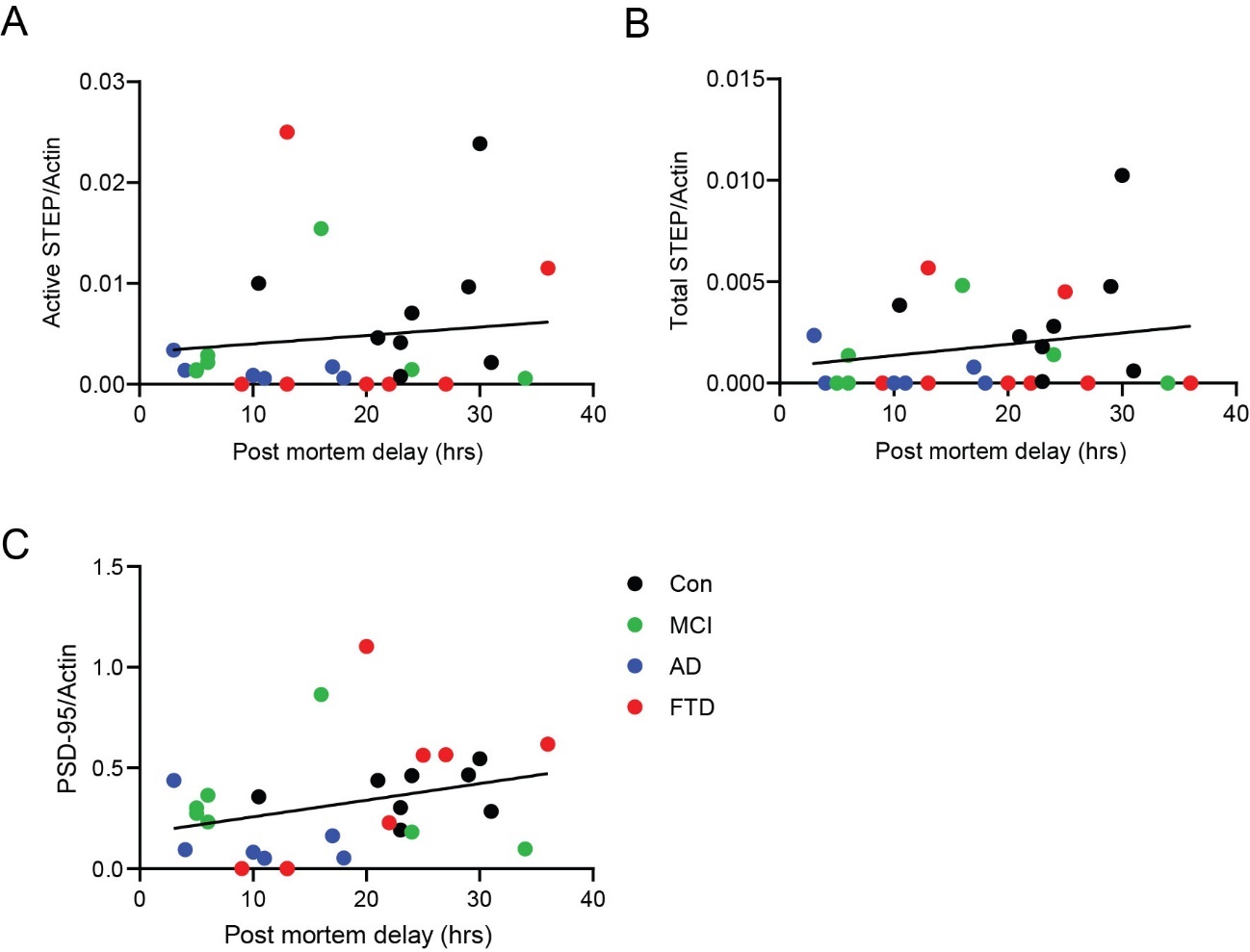


**Supplementary Figure 2. No correlation detected between *post mortem* delay and STEP_61_ activity or expression.**

To determine whether *post mortem* delay effected the phosphorylation state of STEP_61_, as dephosphorylation results in STEP_61_ activation. Long delays can result in degradation of proteins or dephosphorylation by endogenous phosphatases, however we observed no correlation between *post mortem* delay and STEP_61_ activation or expression or PSD-95 expression. Colour coded to show clinical diagnosis of patients. **A** Correlation between active STEP_61_ expression and delay (hrs) of all patients. Correlation analysis by Pearson’s correlation coefficient, all groupedR^2^= 0.01425. **B** Correlation between total STEP_61_ expression and delay (hrs) of all patients. Correlation analysis by Pearson’s correlation coefficient, all grouped R^2^= 0.04894. **C** Correlation between total PSD-95 expression and delay (hrs) of all patients. Correlation analysis by Pearson’s correlation coefficient, all grouped R^2^= 0.09331.


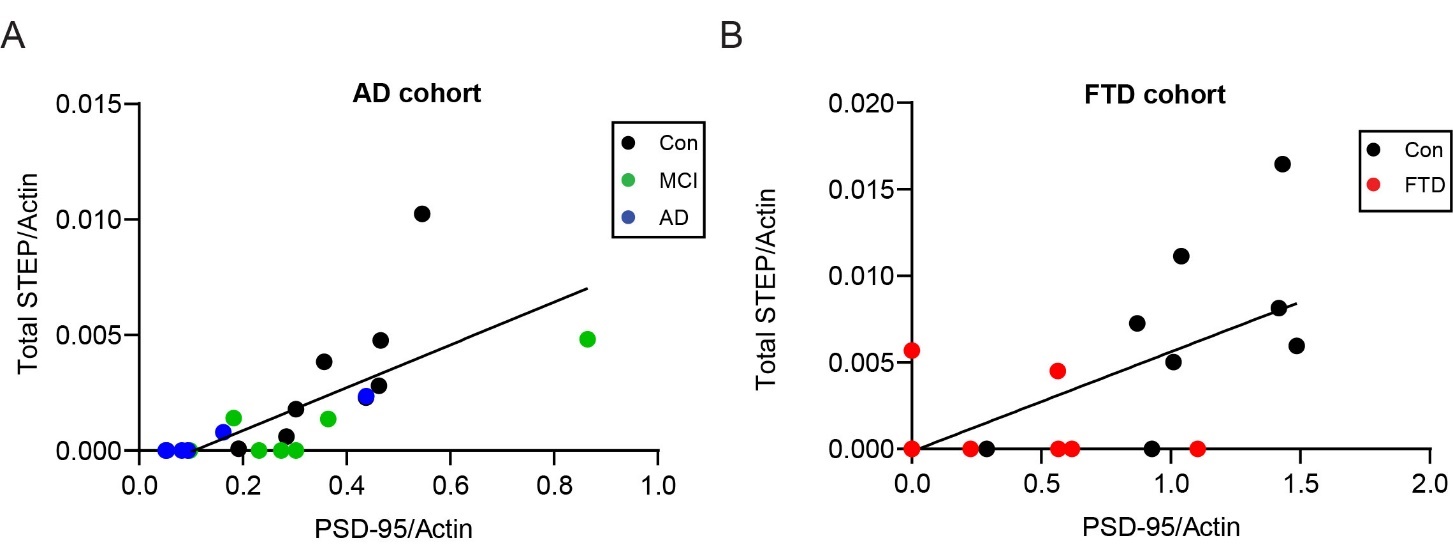


**Supplementary Figure 3. Correlation detected between PSD-95 expression and STEP_61_ expression in AD and FTD cohorts.**

Correlation between the expression of total STEP and PSD-95 expression in AD and FTD human cohorts. Shows decrease in STEP_61_ expression correlates with reduced PSD-95 expression. Colour coded to show clinical diagnosis of patients. **A** Correlation between total STEP_61_ expression and PSD-95 of all AD (blue), MCI (green) and control (black) patients. Correlation analysis by Pearson’s correlation coefficient, R^2^= 0.5297. **B** Correlation between total STEP_61_ expression and PSD-95 of all FTD-tau (red) and control (black) patients. Correlation analysis by Pearson’s correlation coefficient, R^2^= 0.3604.


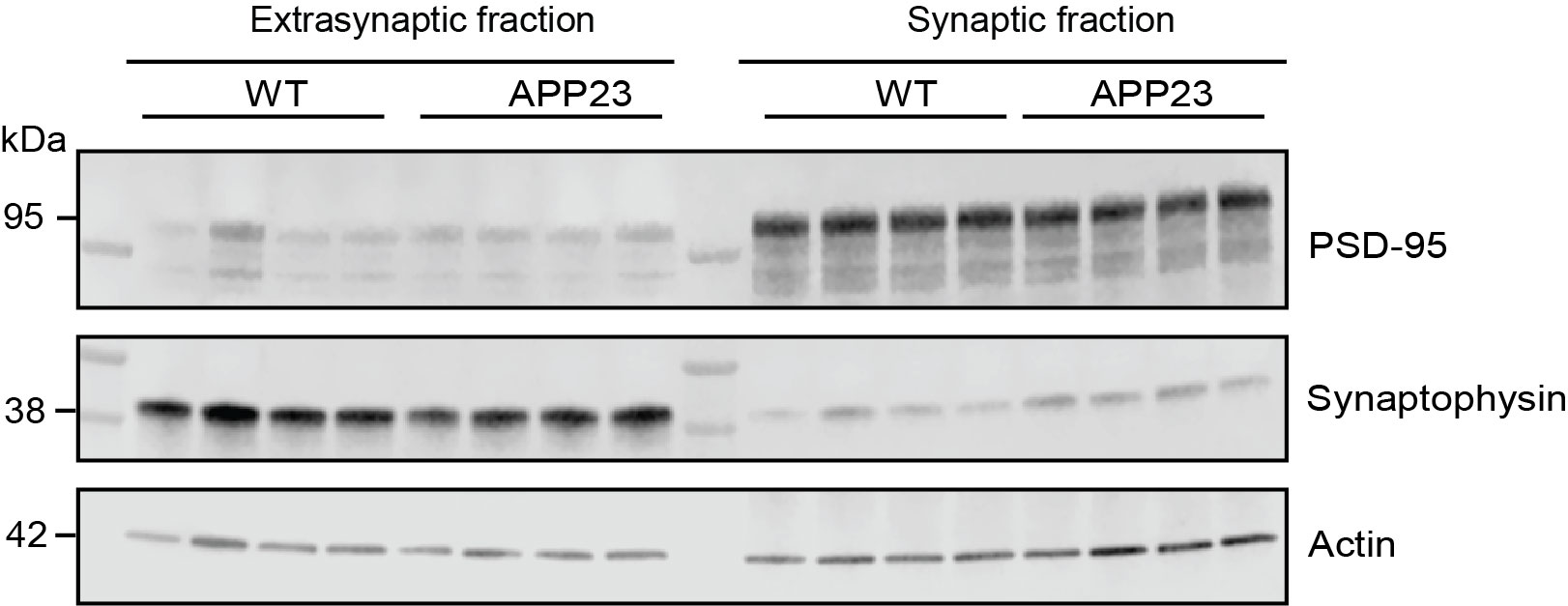


**Supplementary Figure 4. Fractionation protocol quality was identified by extrasynaptic enriched synaptophysin and synaptic enriched PSD-95.**

Immunoblot of the extrasynaptic and synaptic fractions of 6-month-old APP23 mice (n=4) and their WT littermate controls (n=4). Extrasynaptic fractions were enriched for synaptophysin, whereas the synaptic fraction was enriched with the post-synaptic marker PSD-95.
